# Energy of a Sheet of Paper

**Published:** 1885-03

**Authors:** 


### THE ENERGY OF A SHEET OF PAPER.

The modern cargo steamer has now become a wonderfully economical freight carrier, especially as regards consumption of fuel. A freight train run under the most favorable conditions seems wasteful in comparison. The "Burgos," a modern steamer, built to carry cargo cheaply at a slow speed, lately left England for China wirh a cargo weighing 5,600,000 pounds. During the first part of the voyage from Plymouth to Alexandria the consumption of coal was 282,240 pounds, the distance being 3,380 miles. The consumption per mile was therefore only 83.5 pounds, and the consumption per ton of cargo per mile was 0.028 pound. In other words, half an ounce of coal propelled one ton of cargo one mile. Assuming that paper is as efficient a fuel as coal, we have, says the *Railroad Gazette*, only to burn a letter on board this steamer to generate and utilize enough energy to transport one ton of freight one mile.

